# Working status and risk of Alzheimer's disease: A Mendelian randomization study

**DOI:** 10.1002/brb3.2834

**Published:** 2022-12-07

**Authors:** Jiaxi Zhao, Kaixin Li, Xiaoyang Liao

**Affiliations:** ^1^ Department of General Practice, General Practice Medical Center, West China Hospital, West China School of Medicine Sichuan University Chengdu Sichuan China; ^2^ Department of Nephrology Huadong Hospital Affiliated to Fudan University Shanghai China

**Keywords:** Alzheimer's disease, job involves heavy manual or physical work, job involves mainly walking or standing, job involves shift work, Mendelian randomization

## Abstract

**Background:**

Alzheimer's disease (AD) has become a common illness affecting the elderly, adding to society's social and financial burden. We used two‐sample Mendelian randomization (MR) in this study to determine the association between working status and AD.

**Methods:**

We performed a two‐sample MR analysis. The genetic associations were derived from the UK Biobank (*n* = 263,615) and the International Genomics of Alzheimer's Project (*n* = 63,926). Inverse variance weighted (IVW), MR‐Egger, and weighted median were used in the MR analysis. The funnel plot, Cochran's *Q* test, MR‐Egger intercept test, and leave‐one‐out analysis were used in sensitivity analyses. Further risk factor analyses were carried out to look into the potential mediators.

**Results:**

Jobs involve heavy manual or physical work (OR = 2.13, 95%CI 1.36–3.36; *p* = .0011), job involves mainly walking or standing (OR = 1.74, 95%CI 1.19–2.54; *p* = .004), and job involves shift work (OR = 2.78, 95%CI 1.14–6.80; *p* = .02) increased the risk of AD in the IVW analysis. There was no heterogeneity and no horizontal pleiotropy in the sensitivity analysis. Risk factor analysis suggested that each of the above association may be mediated by different risk factors.

**Conclusion:**

Our study adds to the evidence that the development of AD is associated with the working status (job involves heavy manual or physical work, job involves mainly walking or standing, and job involves shift work) by using extensive human genetic data.

## Graphical abstract

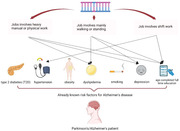

## 1 INTRODUCTION

With a rising incidence, Alzheimer's disease (AD) has become a frequent illness affecting the elderly, adding to the disease's social and financial burden (GBD 2019 Dementia Forecasting Collaborators, 2022; Prince et al., 2016). However, the available data revealed that there were no effective therapy options. Additionally, it was difficult to identify and diagnose AD in its early stage. Therefore, finding preventative strategies is especially important. Previous epidemiological studies have pointed out some potentially modifiable risk factors as preventive measures to reduce the incidence of AD (Larsson et al., 2017; Slomski, 2022). These factors include type 2 diabetes (T2D), hypertension, dyslipidemia, obesity, depressive status, physical inactivity, smoking, and low education. However, there were no studies on the association between working status and AD.

The growth of the modern economy has increased people's work‐related stress, which may be harmful to their physical and mental health (Eng et al., 2022; Lasalvia & Tansella, 2011). For instance, high job intensity and stress may increase employees’ risk of depression (Almroth et al., 2021), and work‐related stress might increase the risk of cardiovascular disease (CVD) (Popovic et al., 2022). Studies on the link between work and disease are, however, exceedingly scarce because neither working stress nor working status could be precisely assessed due to individual subjectivity. To date, working status and AD development have not been thoroughly explored, and the causal relationship between working status and AD development remains unclear.

Potential confounding and reverse causation have an impact on traditional observational studies’ capacity to infer causality (Davey & Hemani, 2014). Mendelian randomization (MR) is the process of identifying and measuring the causal link between exposure and outcome by using genetic variation as an instrumental variable (IV) (Carter et al., 2021). The MR study utilized summary statistics from genome‐wide association studies (GWASs) rather than individual‐level data.

Using the statistics of working status and AD from GWAS, we employed a two‐sample MR in this study to determine the causal link between working status and AD.

## 2 METHODS

### 2.1 Study design and basic assumptions of MR

This is a two‐sample MR study to investigate the causal relationship between three work‐related phenotypes and AD.

MR requires three strict assumptions to be satisfied. First, these IVs are strongly correlated with working status. Second, the IVs is independent of the confounding factor between working status and AD. Third, the IVs has no direct effect on AD, it only affects the outcome through working status.

### 2.2 Work‐related phenotypes

The GWAS of work‐related phenotypes were derived from the UK Biobank, which is a large biomedical database of 500,000 UK participants, accessible globally to approved researchers (Sudlow et al., 2015). The work‐related phenotypes were job involves heavy manual or physical work (sample size = 263,615), job involves mainly walking or standing (sample size = 263,556), and job involves shift work (sample size = 263,315). These phenotypes were all self‐reported categorical variables. We identified single‐nucleotide polymorphisms (SNPs) that were associated with each work‐related phenotype, and genome‐wide significance was defied as *p* < 5 × 10^−8^. We identified 25 SNPs associated with job involves heavy manual or physical work, 16 SNPs with job involves mainly walking or standing, and six SNPs with job involves shift work. None of the SNPs were in linkage disequilibrium in any of the phenotypes (*r* ^2^ < 0.001). Then, to guarantee the strength of the genetic instruments and mitigate the potential effects of weak instrument bias, we filtered SNPs with *F*‐statistics > 10. And we excluded ambiguous and palindromic SNPs by the harmonizing process. Finally, 20, 14, and six SNPs were used as IVs for job involves heavy manual or physical work, job involves mainly walking or standing, and job involves shift work, respectively. The overview of GWAS datasets for work‐related phenotypes and corresponding SNPs were listed in Additional Files S1 and S3–S5. And the data source can be found in https://www.medrxiv.org/content/10.1101/2021.06.28.21259622v1.full.

### 2.3 Alzheimer's disease

A large GWAS meta‐analysis of non‐Hispanic Whites (21,982 cases, 41,944 cognitively normal controls) from the International Genomics of Alzheimer's Project (IGAP) revealed genetic associations with AD. IGAP was composed of four consortia: Alzheimer Disease Genetics Consortium, Cohorts for Heart and Aging Research in Genomic Epidemiology Consortium, The European Alzheimer's Disease Initiative, and Genetic and Environmental Risk in AD/Defining Genetic, Polygenic and Environmental Risk for Alzheimer's Disease Consortium. The GWAS data and the detail were published elsewhere(https://doi.org/10.1038/s41588‐019‐0358‐2). The overview of GWAS datasets for AD was listed in Additional File S2. The genome‐wide summary statistics (registration number NG00075) were obtained by applying to the National Institute of Alzheimer's Disease Genetics of Aging Data Storage Site (NIAGADS).

### 2.4 MR analyses

After harmonization of the effect alleles across the GWASs of working status and AD, we used three MR analysis methods in this study to assess the causal effect of working status on AD. As the primary analysis, we used standard inverse variance weighted (IVW) estimates (Burgess & Thompson, 2017), because this analysis assumes that the tool can only affect the results through exposure and not through any other means. In order to maintain the robustness of the results, MR‐Egger regression and weighted median were used as supplements to IVW (Bowden et al., 2015). MR‐Egger regression and weighted median can be used in a wider range of scenarios, but are less efficient (CIs are wider).

### 2.5 Sensitivity analysis

The Cochran's *Q* statistics, funnel plots, leave‐one‐out analyses, and MR‐Egger intercept tests were performed in the sensitivity analysis. Heterogeneity was present if the *p* value < .05 in Cochran's Q statistic and the results was visualized by the funnel plots. In the leave‐one‐out analysis, we discarded one exposure‐related SNP at a time and repeated the IVW analysis to assess the robustness of the results. Horizontal pleiotropy is a condition in which IVs associated with the exposure (working status) influence the outcome (AD) via multiple factors other than the exposure. To avoid this, we performed the MR‐Egger intercept tests to assess the impact of potential pleiotropic effects. Heterogeneity was present if the *p* value < .05.

### 2.6 Risk factors analysis

We used MR methods to assess the association between working status and currently known risk factors for AD in order to further investigate potential intermediate mediators between working status and increased AD risk. These potential risk factors include T2D, hypertension, obesity, dyslipidemia, smoking, depression (Larsson et al., 2017; Slomski, 2022). MR was used, with working status as the exposure factor and the potential risk factors listed above as the outcome. *p* < .05 for IVW was considered significant. The overview of the GWAS data for the above risk factors was listed in Additional File S6.

### 2.7 Statistical analysis

The MR estimates were represented by odds ratios (ORs) and corresponding 95% confidence intervals (CIs), as well as the estimated change in the relative risk of AD for each standard deviation (SD) increase in working status. All statistical analyses were performed using TwoSampleMR package (version 0.5.6) in R (2022.02.3) (Hemani et al., 2018).

## 3 RESULTS

### 3.1 MR analyses

According to IVW analysis, jobs involve heavy manual or physical work increased the risk of AD (OR = 2.13, 95%CI 1.36–3.36; *p* = .0011). In weighted median methods, it also increased the risk of AD (OR = 2.89, 95%CI 1.48–5.63; *p* = .0018). MR‐Egger showed a consistent direction, but not significant results. Job involves mainly walking or standing was significantly associated with an increased risk of AD (OR = 1.74, 95%CI 1.19–2.54; *p* = .004) in the IVW analysis. Results from weighted median and MR‐Egger methods were in the same direction with IVW, but not statistically significant. Job involves shift work was significantly associated with an increased risk of AD (OR = 2.78, 95%CI 1.14–6.80; *p* = .02) in the IVW analysis and weighted median methods(OR = 4.12, 95%CI 1.27–13.30; *p* = .02), respectively. MR‐Egger showed no significant results (Additional File S7). The results remained statistically significant adjusted for intelligence and education level (Additional File F2).

### 3.2 Sensitivity analysis

**FIGURE 1:**
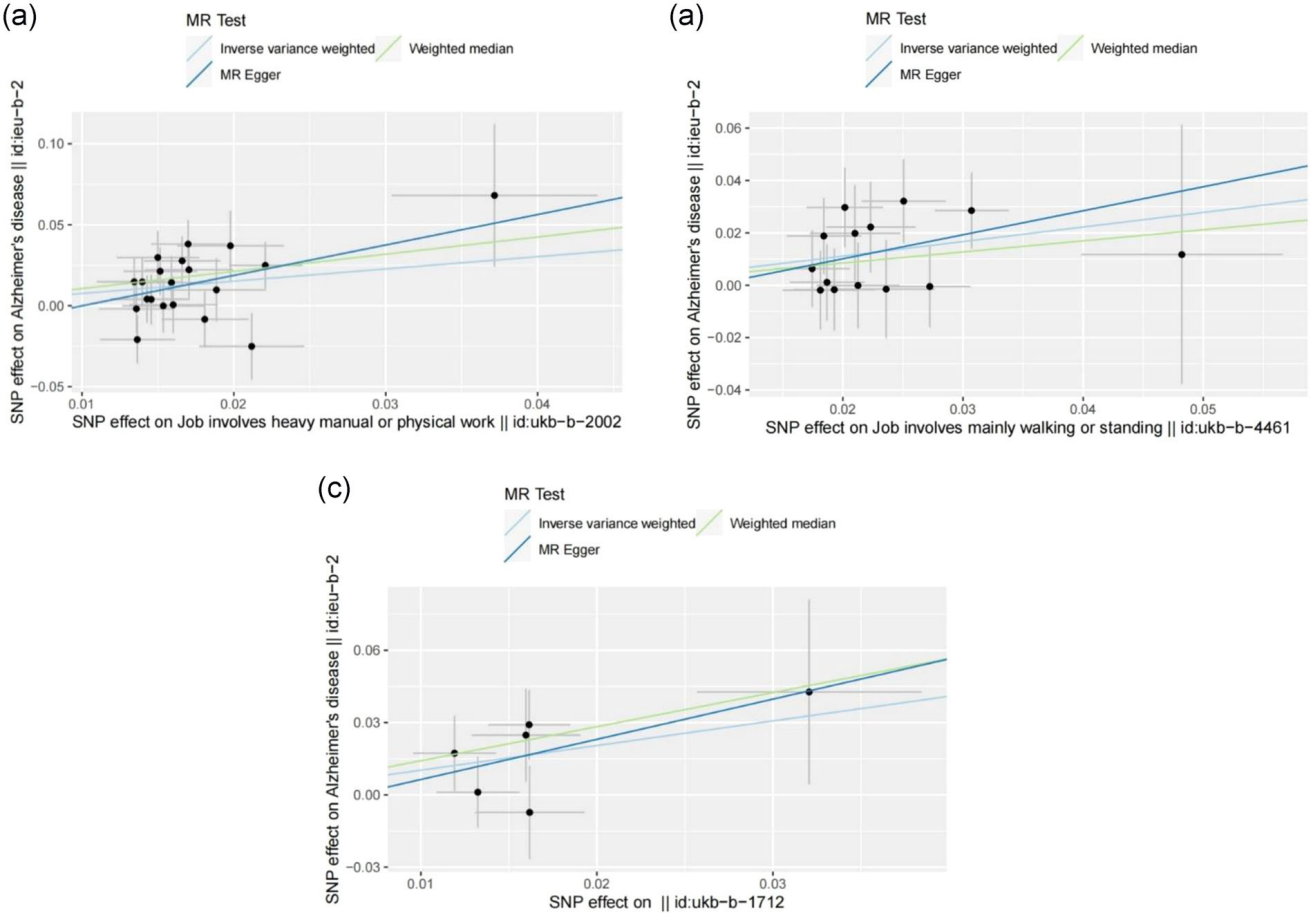
Causal relationships between working status phenotypes and Alzheimer's disease in scatterplots. (A) Jobs involve heavy manual or physical work and Alzheimer's disease. (B) Jobs involve mainly working or standing and Alzheimer's disease. (C) Jobs involve shift work and Alzheimer's disease.

**FIGURE 2:**
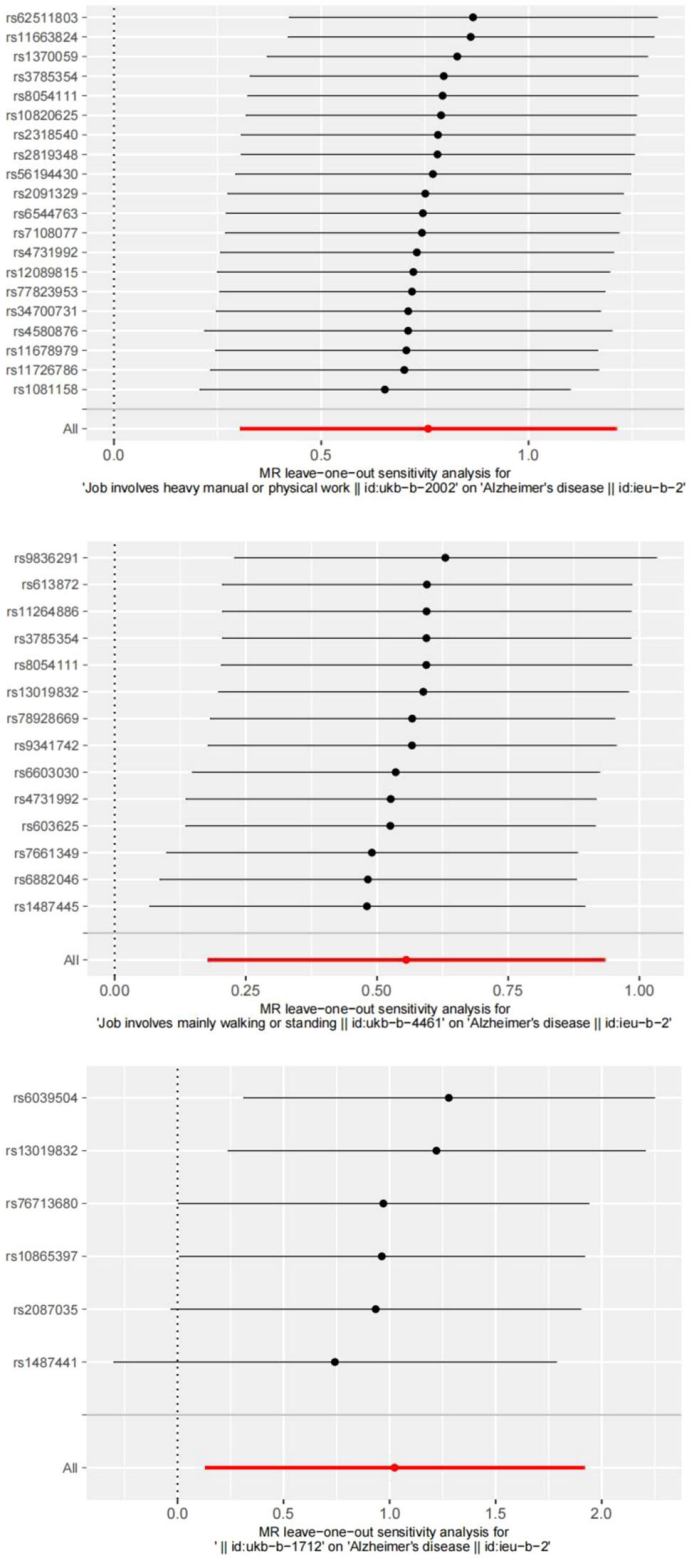
Forest plots of SNPs associated with working status phenotypes and risk of Alzheimer's disease. SNP, single‐nucleotide polymorphism.

We further conducted funnel pot, Cochran's *Q* test, leave‐one‐out analyses, and MR‐Egger intercept tests in the sensitivity analyses. For job involves heavy manual or physical work, job involves mainly walking or standing, and job involves shift work, all *p* values for the Cochran's *Q* test analysis were more than 0.05, indicating no heterogeneity (Table 1). The funnel pot was shown in the Additional File S1. All *p* values for the MR‐Egger intercept test were more than 0.05, indicating no horizontal pleiotropy (Figure 1). The leave‐one‐out analyses showed the robustness of the results (Figure 2).

**TABLE 1:** Sensitivity analysis of the causal association between working status and the risk of Alzheimer's disease

Exposure	Outcome	Cochran *Q* test		MR‐Egger	
		*Q* value	*p* Value	Intercept	*p* Value
Job involves heavy manual or physical work	Alzheimer's disease	20.98	.338	−0.0188	.384
Job involves mainly walking or standing		8.30	.823	−0.0083	.701
Job involves shift work		3.42	.635	−0.0103	.749

### 3.3 Risk factor analysis

**FIGURE 3:**
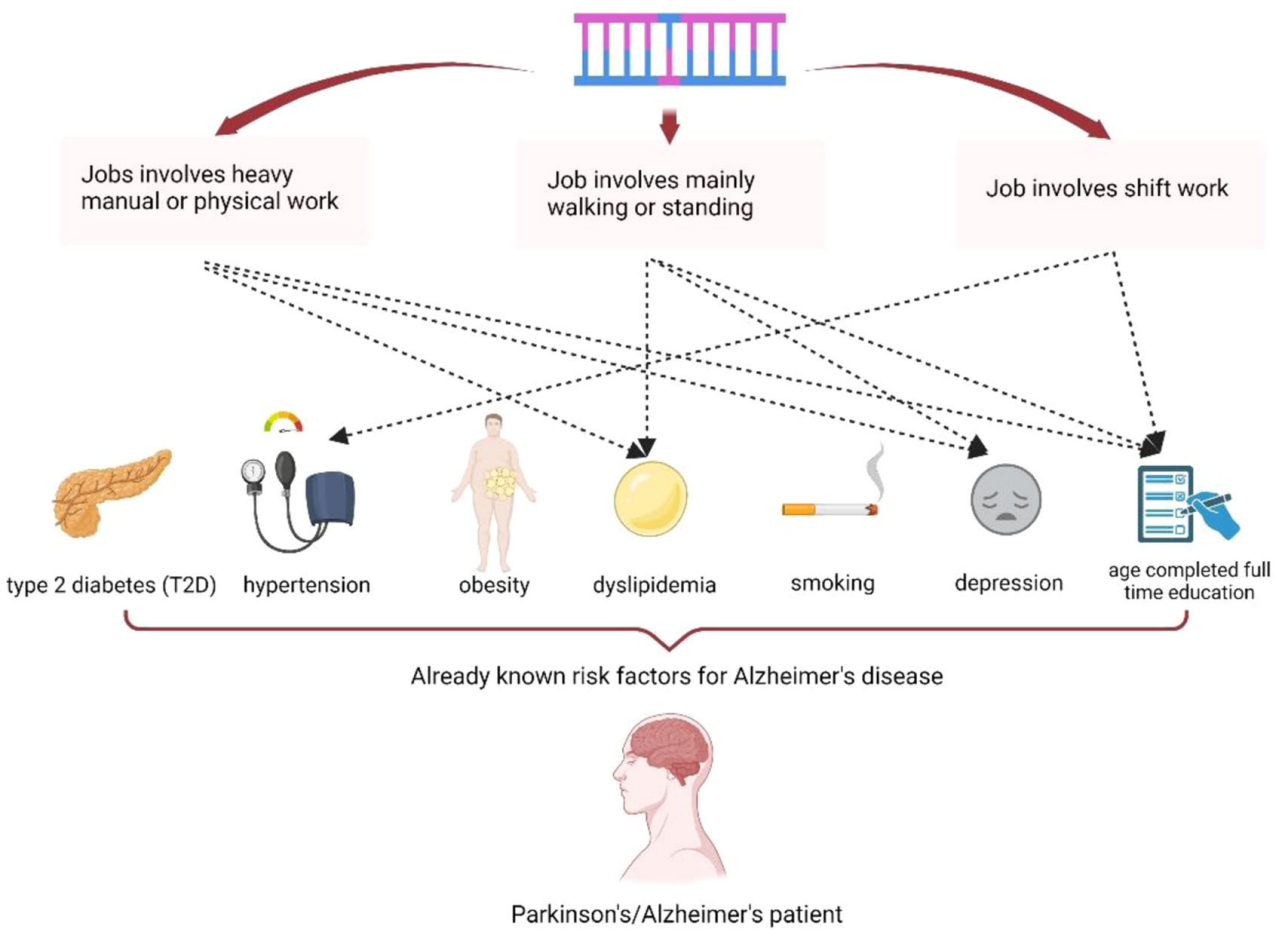
Overview of the MR and risk factor analysis.

To further explore potential mediators linking working status to an increased risk of AD, we set working status as exposures and various risk factors as outcomes. Dyslipidemia (OR = 1.00, 95%CI = 1.00–1.01, *p* = .017), smoking(OR = 1.14, 95%CI = 1.08–1.21, *p* = 2.993× 10^−6^) and age at completion of full‐time education (OR = 0.59, 95%CI = 0.54–0.63, *p* = 6.333× 10^−46^) might be responsible for job involves heavy manual or physical work‐linked AD susceptibility. Furthermore, job involves mainly walking or standing were associated with dyslipidemia (OR = 1.01, 95%CI = 1.00–1.02, *p* = .032), smoking (OR = 1.07, 95%CI = 1.01–1.13, *p* = .017) and age at completion of full‐time education (OR = 0.69, 95%CI = 0.64–0.75, *p* = 6.361×10^−18^). And hypertension (OR = 1.03, 95%CI = 0.99–1.06, *p* = .113) and age at completion of full‐time education (OR = 0.51, 95%CI = 0.42– 0.62, *p* = 1.040× 10^−11^) might be responsible for job involves shift work‐linked AD susceptibility (Table 2 and Figure 3).

**TABLE 2:** Risk factor analysis of working status and currently known risk factors for Alzheimer's disease

Exposure	Outcome	IVW		Cochran *Q* test		MR‐Egger	
		OR(95%CI)	*p* Value	*Q* value	*p* Value	Intercept	*p* Value
Job involves heavy manual or physical work	Type 2 diabetes	0.71(0.24–2.03)	.519	27.01	.255	0.0413	.402
	Hypertension	1.03(0.99–1.07)	.053	86.58	2.653× 10^−9^	0.0002	.899
	Obesity	1.00(0.99–1.01)	.616	57.12	5.893× 10^−5^	0.0002	.525
	Dyslipidemia	1.00(1.00–1.01)	.017*	35.42	.025	−0.0005	.099
	Smoking	1.14(1.08–1.21)	2.993× 10^−6^	75.69	1.559× 10^−7^	0.0008	.731
	Depression	1.01(0.99–1.02)	.545	37.14	.032	−0.0002	.831
	Age at completion of full‐time education	0.59(0.54–0.63)	6.333× 10^−46^*	41.11	.012	−0.0045	.145
Job involves mainly walking or standing	Type 2 diabetes	1.25(0.43–3.66)	.682	18.50	.185	−0.0032	.960
	Hypertension	1.02(0.98–1.06)	.293	64.48	1.894× 10^−8^	0.0005	.796
	Obesity	1.00(0.99–1.02)	.204	39.95	7.323× 10^−5^	−8.4245× 10^−5^	.901
	Dyslipidemia	1.01(1.00–1.02)	.032*	30.92	.002	−0.0005	.288
	Smoking	1.07(1.01–1.13)	.017*	49.37	7.793× 10^−6^	0.0015	.596
	Depression	0.99(0.98–1.01)	.557	18.51	.185	−0.0007	.344
	Age at completion of full‐time education	0.69(0.64–0.75)	6.361× 10^−18^*	34.97	.001	−0.0013	.767
Job involves shift work	Type 2 diabetes	4.27(0.39–0.47)	.234	3.65	.455	−0.1211	.442
	Hypertension	1.05(1.01–1.10)	.027*	6.329	.275	0.0002	.925
	Obesity	1.01(0.98–1.03)	.677	15.01	.005	0.0003	.879
	Dyslipidemia	1.01(0.99–1.04)	.298	21.33	2.726× 10^−4^	0.0011	.578
	Smoking	1.03(0.96–1.10)	.448	3.44	.633	0.0015	.564
	Depression	1.03(0.99–1.06)	.113	6.99	.222	0.0010	.475
	Age at completion of full‐time education	0.51(0.42–0.62)	1.040× 10^−11^*	0.11	.736	0.0012	.456

IVW, inverse variance weighted; OR, odds ratio; 95%CI, 95% confidence interval.

## 4 DISCUSSION

This study utilized large‐scale GWAS data from the UK Biobank and the International Genomics of Alzheimer's Project. And our study employed multiple MR methods to explore the possible association between working status and AD susceptibility. It was found that job involves heavy manual or physical work, job involves mainly walking or standing, and job involves shift work increased the risk of AD. AD is a high‐incidence disease with insidious onset, and lack of prevention and treatment options, and our study offers fresh insights towards lowering the risk of AD by stressing the link between working status and AD.

With the increased emphasis on health in modern times, understanding how to maintain a healthy lifestyle is important, and these modifiable lifestyles influence whether or not each of us suffers from the disease (Jin, 2022; Kucuk, 2022). Many aspects of lifestyle are discussed, including eating habits, physical activities, smoking, and drinking alcohol. However, because of the work's high flexibility and the relatively low feasibility of relevant clinical trials, less attention has been paid to the health effects of work on people. A meta‐analysis of Li et al. (2021) showed that working stress was an important risk factor for T2DM, especially in women. The most important mechanism might be neuroendocrine dysregulation, which includes sympathetic nerve activation and dysregulation of the hypothalamic–pituitary–adrenal axis (HPA) (Mcewen, 2006, 2008). According to Eshak et al. (2022), jobs involving physical labor were associated with lower colon cancer risk compared with office work, and moving regularly during work was associated with a lower risk of rectal cancer compared with sitting. Furthermore, working status and stress were linked to an increased risk of cardiovascular and cerebrovascular diseases, but there was currently no research on the relationship between working status and AD. Ours was the first study to use MR methods to investigate the association between working status and AD.

Although the mechanism by which working status increases AD susceptibility remains unknown, dyslipidemia, smoking, hypertension, and education level are known risk factors for AD. High cholesterol levels may be linked to an increased risk of AD (Xue‐Shan et al., 2016), and higher total cholesterol and LDL‐C concentrations are associated with faster cognitive decline in AD patients (Kao et al., 2020). Previous research has suggested that taking statins early and for a long time might protect against dementia, particularly vascular dementia (Kim et al., 2021). The mechanisms linking dyslipidemia to AD include changes in the gut‐brain axis, mitochondrial dysfunction, oxidative stress, and inflammation, all of which lead to synaptic loss and, eventually, cognitive decline (Kao et al., 2020). A recent meta‐analysis of prospective studies showed that current smokers have a higher risk of developing AD than never smokers, implying that smoking is a risk factor for AD (Peters et al., 2008). However, early reports suggested that smoking might be protective against AD due to nicotine's neuroprotective effects (Salomon et al., 1996), though this association may be due to some bias. Hypertension is considered a risk factor for AD (Barnes & Yaffe, 2011). However, this association remains uncertain and may be complicated by misclassification of AD from other forms of dementia. Some findings suggest that high blood pressure in midlife is associated with a higher risk of AD, but others suggest that AD may be protected by high blood pressure later in life (Launer et al., 2000; Malik et al., 2021). As for the association between education level and AD, previous research results were controversial. A 2011 cross‐sectional study showed that the higher the education level, the lower the risk of AD (Barnes & Yaffe, 2011). A 2015 MR analysis did not find the causal association (Ostergaard et al., 2015).

Whether job involves heavy manual or physical work, job involves mainly walking or standing, or job involves shift work have an effect on working pressure, and each has its characteristics, for example, job involves heavy manual or physical work might involve a lot of anaerobic exercise. Although physical activity and exercise have been shown to be effective strategies for the prevention and treatment of dyslipidemia (Kruger et al., 2022), the negative health effects of mental stress may be more pronounced because work requires both physical and mental commitment. A previous study found that working stress increased risk of dyslipidemia by affecting the HPA (Zhang et al., 2021), and chronic cortisol elevation increases glucocorticoid synthesis and glucose utilization, increases visceral fat deposition, and accelerates lipolysis, resulting in dyslipidemia (Kao et al., 2020). Additionally, working stress also affects smoking habits, a cross‐sectional study of the Asian population showed that working stress was significantly associated with smoking((OR = 1.45, 95%CI = 1.17–1.80) (Cheng et al., 2022). Shift work is the basic form of enterprise working time organization, which refers to the formation of work groups of different shifts within the working day, in the same work place for the production process. A meta‐analysis of five cohort studies showed that a 5‐year increase in shift work was associated with a 5% increased risk of CVD (Wang et al., 2018). A cohort study among nurses found that those with 10 or more night shifts per month were significantly more likely to have high blood pressure (Zhao et al., 2021), the potential mechanism might be that shift work leads to circadian disruption, systemic inflammation, and decreased melatonin production (Folkard, 2008). It is generally known that the different job is affected by the education level. Those with high education level are more likely to engage in intellectual work, while those with low education level are more likely to engage in physical work. However, the mechanism of working status’ impact on education level is unknown.

However, this study still has several limitations. First, the GWAS data for this study were derived from participants of European ancestry and so generalization to other populations with different cultural traditions and lifestyles is limited. Second, there was some subjectivity in the working status, and there were differences in different people's understanding of working status and intensity, which could have an impact on the research results. Third, given the limitations of the UK Biobank data, future studies should delve deeper into causal relationships and underlying mechanisms, which are essential for clinical application. Furthermore, the exact biological function of many genetic variants remains unknown. And there are still some risk factors for AD that need to be identified. The pathophysiology of the relationship between working status and AD needs to be further studied. In the future, more intervention and cross‐sectional studies are needed to explore the relationship between working status and AD in the real world.

In conclusion, using large‐scale human genetic data, our study further strengthens the evidence that working status is linked to the development of AD. Further research is needed to explore the underlying mechanisms between working status and AD. In view of the difficulty of prevention and treatment of AD, more attention should be paid to discover modifiable risk factors to reduce the susceptibility of AD.

## AUTHOR CONTRIBUTION

*Conceptualization, methodology, formal analysis, writing, and visualization*: Jiaxi Zhao. *Validation, data curation, and visualization*: Kaixin Li. *Supervision and project administration*: Xiaoyang Liao.

## CONFLICT OF INTEREST

The authors declared that they have no conflicts of interest to this work. We declare that we do not have any commercial or associative interest that represents a conflict of interest in connection with the work submitted.

### RESOURCE IDENTIFICATION

We used GWAS data from UK Biobank (UK Biobank, RRID: SCR_012815) and we used GWAS data from NIAGADS (NIAGADS, RRID: SCR_007314).

Alzheimer's disease. A large GWAS meta‐analysis of non‐Hispanic Whites (NHW) (21,982 cases, 41,944 cognitively normal controls) from the International Genomics of Alzheimer's Project (IGAP) revealed genetic associations with AD. IGAP was composed of four consortia: Alzheimer Disease Genetics Consortium, Cohorts for Heart and Aging Research in Genomic Epidemiology Consortium, The European Alzheimer's Disease Initiative, and Genetic and Environmental Risk in AD/Defining Genetic, Polygenic and Environmental Risk for Alzheimer.s Disease Consortium. The GWAS data and the detail were published elsewhere (doi: 10.1038/s41588‐019‐0358‐2). The overview of GWAS datasets for Alzheimer's disease was listed in Additional file S2. The genome‐wide summary statistics (registration number NG00075, RRID: SCR_007314) were obtained by applying to the National Institute of Alzheimer's Disease Genetics of Aging Data Storage Site (NIAGADS).

### PEER REVIEW

The peer review history for this article is available at https://publons.com/publon/10.1002/brb3.2834.

## Supporting information

Supporting material

## Data Availability

All data in the article can be obtained by sending an email to the author.
